# A Patient with Multiple Gastrointestinal Carcinoid Tumours Presenting with Jejunal Intussusception

**DOI:** 10.1155/2021/5525086

**Published:** 2021-02-17

**Authors:** Kavinda Deshapriya Bandara, Sanjeevan Ravindrakumar, Kirushanthan Veerasingam, Umesh Jayarajah, V. S. D. Rodrigo

**Affiliations:** Department of Surgery, District General Hospital Chilaw, Chilaw, Puttalam District, Sri Lanka

## Abstract

Carcinoid tumours are neuroendocrine tumours which arise from the enterochromaffin cells in the gastrointestinal and bronchopulmonary systems. The presentation of multiple gastrointestinal carcinoids with jejunal intussusception is rare, and the diagnosis may be challenging. A 49-year-old patient with adult onset bronchial asthma presented with pain around the umbilical region for 1-day duration. Physical examination revealed only mild abdominal tenderness. Abdominal computed tomography revealed small bowel intussusception with two separate highly vascular tumours arising in the small bowel mesentery. Exploratory laparotomy was done, with resection of the tumours arising from the small bowel mesentery and the proximal jejunum causing the intussusception which were excised. Histopathological diagnosis confirmed the presence of a Grade 1 carcinoid tumour of classic type. After surgery, he had an uneventful recovery and was asymptomatic. Carcinoid tumours are a very rare cause of adult intussusception. So far, there have been only two reported cases of jejunal intussusception secondary to carcinoid tumours. These will require a combination of surgical intervention and systemic therapy in selective cases for complete management.

## 1. Introduction

Carcinoid tumours are neuroendocrine tumours which arise from the enterochromaffin cells in the gastrointestinal and bronchopulmonary systems. These rare and slow growing tumours may present with vague symptoms or may be incidentally identified in a laparotomy done for a different indication [1]. Approximately 10% of these tumours will produce vasoactive substances giving rise to the characteristic carcinoid syndrome [2].

Intussusception is another rare occurrence in adults, accounting for 5% of all intussusceptions. They occur mainly in children, and almost all cases of paediatric intussusception are idiopathic. In adults, on the other hand, intussusception is usually secondary to a pathological lead point [3].

In this case report, we present an unusual case of multiple gastrointestinal carcinoid tumours presenting with a jejunal intussusception.

## 2. Case Presentation

A 49-year-old male with adult onset bronchial asthma, on inhalers for the past 5 years, presented with sudden onset severe epigastric pain for 1-day duration. He denied any history of nausea, vomiting, abdominal distension, or a recent change in bowel habits. He has been having similar episodes for the past 2 months and was managed as “gastritis” with proton pump inhibitors. Physical examination revealed a soft, nondistended abdomen, with mild generalized tenderness. Biochemical and haematological investigations were within normal limits.

**Figure 1:**
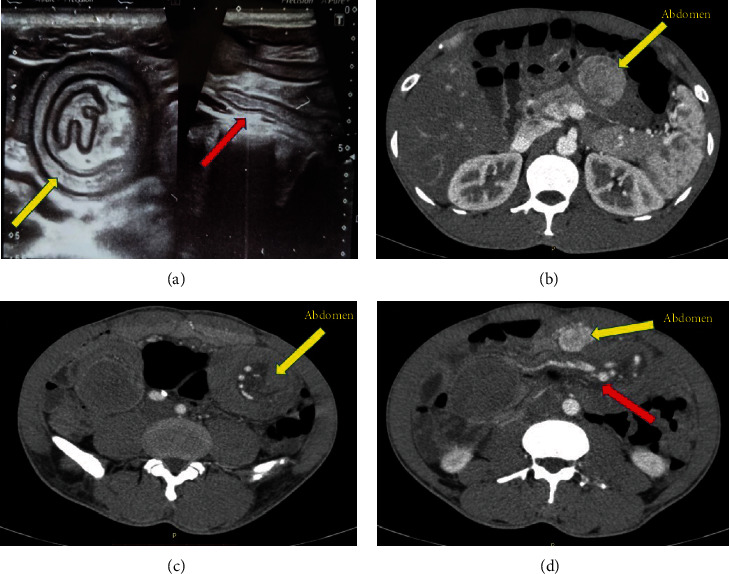
(a) Ultrasound scan showing a small bowel intussusception with the classical target sign (yellow arrow) and longitudinal section shown by red arrow. (b) Contrast enhanced computed tomography (CECT) of abdomen showing a mass (yellow arrow) attached to the proximal mesentery with arterial enhancement. (c) CECT showing proximal bowel intussusception (yellow arrow). (d) CECT showing proximal bowel intussusception (red arrow) and a mass attached to the proximal mesentery (yellow arrow).

Ultrasound scan revealed a small bowel intussusception with the classical target sign (Figure 1). Contrast enhanced computed tomography (CECT) of the abdomen revealed a large segment proximal bowel intussusception, with two intra-abdominal masses with prominent arterial enhancement, attached to the proximal mesentery.

The combination of recent-onset asthma and the highly suggestive radiological findings raised the suspicions a carcinoid tumour. Therefore, serum chromogranin A level was also performed.

**Figure 2:**
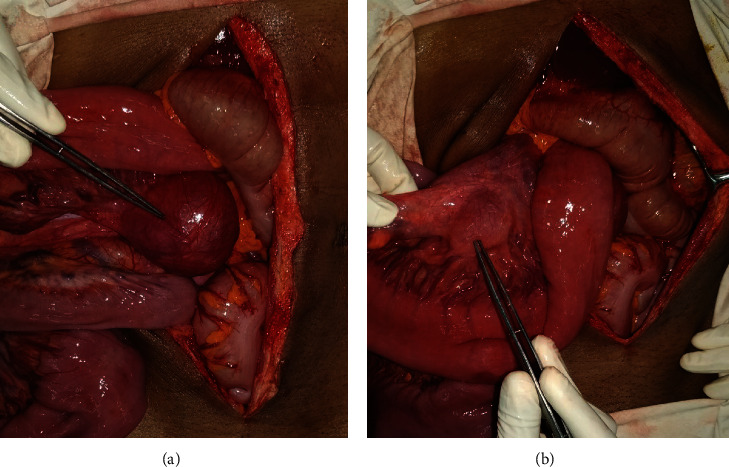
(a, b) Two intra-abdominal masses attached to the proximal mesentery.

**Figure 3:**
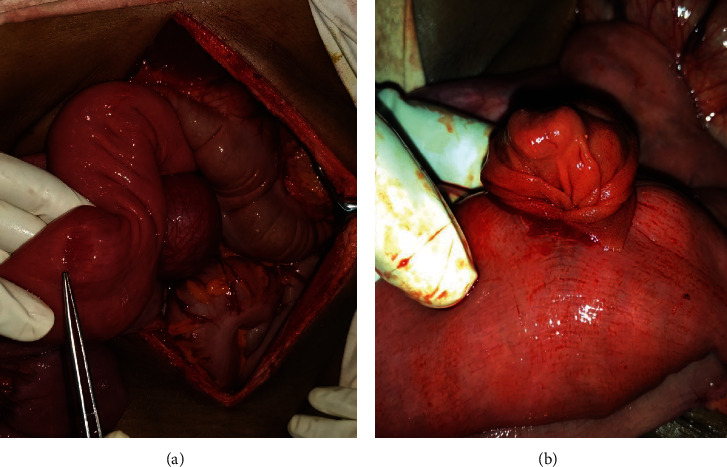
(a, b) A jejunal mass acting as a lead point for intussusception.

The patient underwent an exploratory laparotomy. A proximal jejunal intussusception was identified which was easily reducible. There were three benign-looking tumours, two arising from the proximal small bowel mesentery and a third one arising from the proximal jejunal wall which was the leading point for the intussusception (Figures 2 and 3). The two mesenteric tumours were excised with the vascular pedicle supplying the tumours. The tumour arising from the proximal jejunum was excised with a 1 cm cuff of normal bowel tissue and repaired primarily. The rest of the peritoneal survey was normal, and there was no evidence of liver deposits.

**Figure 4:**
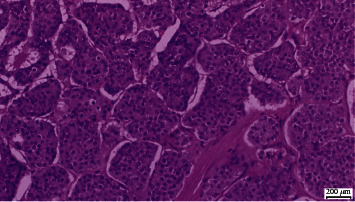
H&E ×40 showing Grade 1 carcinoid tumour of classic type.

Histopathological analyses of all 3 tumours revealed a Grade 1 carcinoid tumour of classic type (Figure 4). The Ki-67 proliferation index was less than 1%. Postoperative period was uncomplicated, and the patient was discharged on day 3. Serum chromogranin A levels that were sent before surgery were high (1093 micrograms/litre (normal < 100)). At 3 months after surgery, serum chromogranin levels were 64.5 (normal < 100). At 6 months of follow-up, the patient was completely asymptomatic and was free of asthmatic episodes.

## 3. Discussion

A typical paediatric case of intussusception usually presents with intermittent colicky abdominal pain, vomiting, and bloody mucoid stools with a palpable abdominal mass. On the other hand, adult intussusception may present with either acute onset, subacute onset, or long-standing symptoms [1, 2]. The reported patient had repeated episodes of acute onset epigastric pain for a 2-month duration. His haematological and biochemical investigations had been normal. Radiological studies had not been performed during the acute episodes of pain, and he was being managed as having “gastritis.” During the index presentation, the ultrasound and CECT scans revealed the classical “target sign,” clinching the diagnosis of intussusception [3]. The multiple enhancing tumours detected by the CECT with late onset asthma in a previously healthy male raised the suspicion of carcinoid tumour. Although upper gastrointestinal endoscopy may be indicated, we did not perform this as the lesions were clearly identified in relation to the proximal bowel in CT imaging. During surgery, thorough inspection and palpation of the entire bowel from the lower oesophagus to the rectum was performed to exclude other synchronous lesions. Furthermore, relief of symptoms without recurrence and normalisation of serum chromogranin levels suggests that any clinically significant residual lesions are unlikely.

Carcinoid tumours are a rare entity of tumours thought to arise from the enterochromaffin (Kulchitsky) cells found in crypts of Lieberkuhn of the gut. Though rare, they are the most common group of gastrointestinal neuroendocrine tumours. In the gastrointestinal tract, nearly 45% of the carcinoid tumours arise in the small intestine, making it the most common site for carcinoid tumours. Of the small bowel, the ileum is the most common site for carcinoid rumors [4, 5]. Carcinoids arising in the jejunum presenting with intussusception are extremely rare and have only been reported twice before [6, 7]. The Table 1 summarises the details of the 2 previously reported cases.

Clinical presentation of the carcinoid tumours varies with their site of origin and their ability to secrete vasoactive substances. Small intestinal carcinoids can give rise to crampy, paroxysmal abdominal pain, bowel obstruction, mesenteric ischaemia, and even lower gastrointestinal bleeding [6].

Approximately, 30-40% of well-differentiated neuroendocrine tumours can produce humoral factors such as polypeptides, vasoactive amines like serotonin (5-HT), and prostaglandins which give rise to carcinoid syndrome. This is characterized by episodic facial flushing accompanied by hypotension and tachycardia, diarrhea, bronchoconstriction, venous telangiectasia, dyspnoea, and ultimately fibrotic complications such as mesenteric and retroperitoneal fibrosis and carcinoid heart disease. However, development of typical carcinoid syndrome is rare in small intestinal tumours accounting only for 5-7%, and they are also associated with the presence of liver metastases [1, 8].

The diagnosis of carcinoid tumours was initially based on histology and was confirmed by positive immunohistochemical staining. A 24-hour urine sampling for 5-hydroxyindoleacetic acid (5-HIAA), an inactive metabolite of serotonin, is highly specific (100%) for diagnosis of carcinoid disease. However, this test lacks sensitivity (73%) as some of the tumours will not secrete serotonin. Serum analysis for chromogranin A levels is also a specific (84-95%) and a more sensitive (75-85%) way of diagnosis as serum chromogranin levels do not rely on the serotonin secretion by the tumour [8].

In cases of adult intussusception, reduction of the intussuscepted segment alone, without resection, is insufficient as there is high risk of the causative lead point being a malignant lesion [3, 9]. Furthermore, the intussuscepted segment should be resected without prior reduction due to the potential for intraluminal seeding or tumour dissemination via lymphatics and veins while manipulating a malignant tumour [9]. Treatment for advanced carcinoid syndrome includes the use of somatosensory analogues (e.g., octreotide and lanreotide), chemotherapy, and vascular endothelial growth factor inhibitors [5].

In our patient, the intussusception was easily reducible, and the jejunal tumour was resected with a wide margin. The timely imaging during the peak of symptoms was key in the diagnosis.

## 4. Conclusion

Intussusception in adults is a rare phenomenon. Majority of the cases are secondary to a structural lesion. Carcinoid tumours are a very rare cause of adult intussusception. The timely imaging during the peak of symptoms is key in the diagnosis. These will require a combination of surgical intervention and systemic therapy in selected cases for complete management. By reporting this case, we hope to contribute to the existing literature.

## Figures and Tables

**Table 1:** Summary of previously reported cases of jejunal intussusception secondary to carcinoid tumours [6, 7].

Author	Case 1	Case 2
	Umashankkar Kannan et al.	Jennifer Matulich et al.
Year reported	2015	2014
Presenting history	Periumbilical abdominal pain for 1 day	Sudden onset diffuse abdominal pain
Past history	Nil to note	Chromic anemia, small bowel resection for ischaemia, volvulus secondary to malrotation, gastric carcinoid tumour (resected endoscopically), and tubular adenoma of colon (polypectomy done)
Examination	Nondistended, soft abdomen with tenderness in umbilical region	Generalized abdominal tenderness
Imaging studies	CT of abdomen showing intussusception in jejunum, without any obvious intra-abdominal masses	CT abdomen showed 3 target signs
Biochemistry	Within normal limits	Within normal limits
Treatment	Exploratory laparotomy done, revealing intussusception in jejunal segment with mesenteric lymphadenopathy and multiple liver nodules	Exploratory laparotomy which revealed an intussusception involving the jejunal segment
Surgery	Resection of involved bowel segment with 5 cm margins of the adjacent unaffected intestine, with subsequent reconstitution of the gastrointestinal tract	Resection of involved jejunal segment followed by jejunojejunal anastomosis
Outcome and follow-up	Recovered well with no postoperative complications	Recovered well with no postoperative complications

## Data Availability

The data used to support the findings of this study are included within the article.

## References

[1] Pinchot S. N., Holen K., Sippel R. S., Chen H. (2008). Carcinoid tumors. *The Oncologist*.

[2] Modlin I. M., Moss S. F., Oberg K. (2010). Gastrointestinal neuroendocrine (carcinoid) tumours: current diagnosis and management. *Medical Journal of Australia*.

[3] Yakan S., Caliskan C., Makay O., Denecli A. G., Korkut M. A. (2009). Intussusception in adults: clinical characteristics, diagnosis and operative strategies. *World Journal of Gastroenterology*.

[4] Bilimoria K. Y., Bentrem D. J., Wayne J. D., Ko C. Y., Bennett C. L., Talamonti M. S. (2009). Small bowel cancer in the United States: changes in epidemiology, treatment, and survival over the last 20 years. *Annals of Surgery*.

[5] Strosberg J. (2012). Neuroendocrine tumours of the small intestine. *Best Practice & research. Clinical Gastroenterology.*.

[6] Kannan U., Rahnemai-Azar A. A., Patel A. N., Gaduputi V., Shah A. K. (2015). Jejunal intussusception: a rare presentation of carcinoid tumor. *Case Reports in Surgery*.

[7] Matulich J., Thurston K., Galvan D., Misra S. (2014). A case of carcinoid likely causing jejunal intussusception. *Case Reports in Surgery*.

[8] Rubin de Celis Ferrari A. C., Glasberg J., Riechelmann R. P. (2018). Carcinoid syndrome: update on the pathophysiology and treatment. *Clinics (Sao Paulo)*.

[9] Barussaud M., Regenet N., Briennon X. (2006). Clinical spectrum and surgical approach of adult intussusceptions: a multicentric study. *International Journal of Colorectal Disease*.

